# Conditioned Medium from Cells Overexpressing TDP-43 Alters the Metabolome of Recipient Cells

**DOI:** 10.3390/cells9102198

**Published:** 2020-09-29

**Authors:** Rudolf Hergesheimer, Débora Lanznaster, Jérôme Bourgeais, Olivier Hérault, Patrick Vourc’h, Christian R. Andres, Philippe Corcia, Hélène Blasco

**Affiliations:** 1 INSERM, UMR 1253, iBrain, Université de Tours, 37000 Tours, France; rudolf.hergesheimer@etu.univ-tours.fr (R.H.); debora.lanznaster@univ-tours.fr (D.L.); patrick.vourch@univ-tours.fr (P.V.); christian.andres@univ-tours.fr (C.R.A.); philippe.corcia@univ-tours.fr (P.C.); 2 CNRS ERL7001, EA 7501 GICC, Université de Tours, 37000 Tours, France; j.bourgeais@chu-tours.fr (J.B.); olivier.herault@univ-tours.fr (O.H.); 3 CHU de Tours, Service de Biochimie et Biologie Moléculaire, 37000 Tours, France; 4 CHU de Tours, Service de Neurologie, 37000 Tours, France

**Keywords:** TDP-43, ALS, prion-like, metabolomics

## Abstract

Amyotrophic lateral sclerosis (ALS) is a neurodegenerative disease caused by the progressive death of both upper and lower motor neurons. The disease presents a poor prognosis, and patients usually die 2–5 years after the onset of symptoms. The hallmark of this disease is the presence of phosphorylated and ubiquitinated aggregates containing trans-active response DNA-binding protein-43 (TDP-43) in the cytoplasm of motor neurons. TDP-43 pathology has been associated with multiple pathways in ALS, such as metabolic dysfunction found in patients and in in vivo models. Recently, it has been described as a “prion-like” protein, as studies have shown its propagation in cell culture from ALS brain extract or overexpressed TDP-43 in co-culture and conditioned medium, resulting in cytotoxicity. However, the cellular alterations that are associated with this cytotoxicity require further investigation. Here, we investigated the effects of conditioned medium from HEK293T (Human Embryonic Kidney 293T) cells overexpressing TDP-43 on cellular morphology, proliferation, death, and metabolism. Although we did not find evidence of TDP-43 propagation, we observed a toxicity of TDP-43-conditioned medium and altered metabolism. These results, therefore, suggest (1) that cells overexpressing TDP-43 produce an extracellular environment that can perturb other cells and (2) that TDP-43 propagation alone may not be the only potentially cytotoxic cell-to-cell mechanism.

## 1. Introduction

Amyotrophic lateral sclerosis (ALS) is a devastating neurodegenerative disease caused by the rapid deterioration of the superior and inferior motor neurons, leading to death of the patient 2–5 years following symptom onset [1]. To this day, no effective treatment exists despite numerous clinical trials. Hence, the continued study of ALS pathogenesis remains critical to better understand the relationship between its pathological mechanisms and motor neuron degeneration. The hallmark of ALS is the presence of phosphorylated and ubiquitinated aggregates containing full-length and cleaved forms of trans-active response DNA-binding protein-43 (TDP-43) in motor neurons [2,3]. The toxicity of this aggregation has been a long-debated subject, but there is increasing evidence arguing that it is tightly related to the neurodegeneration observed in ALS cases [4].

The C-terminus of TDP-43 has been associated with its propensity to aggregate. This region also exhibits a prion-like structure; it is a low-complexity domain, rich in glycine, asparagine, and glutamine residues, and has an undefined tertiary structure. Prions are classified as abnormally folded, transmissible proteins that seed the misfolding of otherwise normally folded copies of itself, usually causing their aggregation and cytotoxicity [5]. In cell culture, “prion-like” seeding and propagation of aggregation have been observed for proteins that are heavily involved in neurodegenerative diseases, such as Parkinson’s, Huntington’s and ALS [6,7,8]. More specifically, researchers have observed this “prion-like” behavior in TDP-43 extracted from diseased brains of ALS and frontotemporal lobar dementia (FTLD) patients [9,10,11], as well as from overexpressed TDP-43 in co-culture [12] and in conditioned medium [13]. This propagation has been associated with cytotoxicity. However, the cellular alterations related to this toxicity have not been fully investigated.

Increasing evidence supports the hypothesis that metabolism is heavily modified in ALS, with reports on hypermetabolism, altered lipid/glucose metabolism, tricarboxylic acid (TCA) cycle, and mitochondrial function [14] in ALS patients. Interestingly TDP-43 has been indirectly linked to metabolic dysfunction. For example, studies have highlighted perturbations in the carnitine shuttle and fatty acid oxidation in mitochondria of transgenic *Drosophila* overexpressing TDP-43 [15], as well as in glycerophospholipid metabolism in a HEK-293T (Human Embryonic Kidney 293T) model [16]. Accordingly, one could suggest an effect of TDP-43 propagation on cellular metabolism, which may be associated with its toxicity.

**Figure 1:**
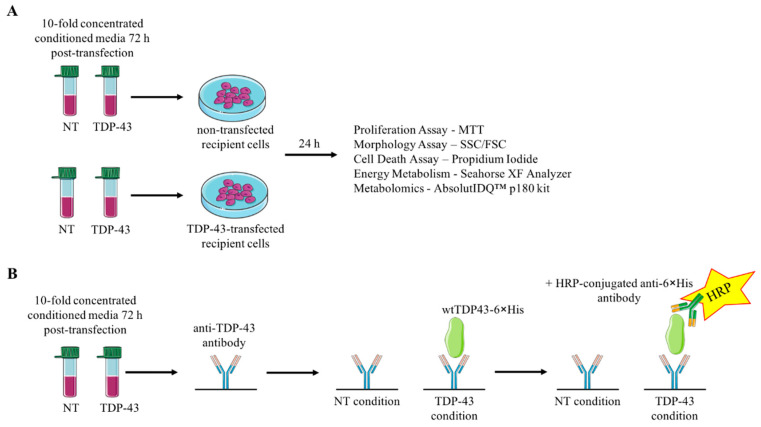
General workflow of the overall study. (**A**) Media from HEK-293T (Human Embryonic Kidney 293T) either non-transfected or transfected with wilde-type trans-active response DNA-binding protein-43 (wtTDP-43) were recovered after 72 h of overexpression. They were concentrated 10-fold by centrifugation and applied to naïve or TDP-43-overexpressing HEK-293T 24 h post-transfection. After 24 h of incubation, the assays mentioned in the figure were performed on the naïve recipient cells. (**B**) Protocol to measure the presence of overexpressed histidine-tagged wtTDP43 (wtTDP-43-6×His) in conditioned medium by ELISA (enzyme-linked immunosorbent assay). Briefly, HEK-293T cells were either treated with transfection agent only or transfected with wtTDP43-6×His cDNA. After 72 h, the media were recovered and concentrated 10-fold. These media were then applied to the sandwich ELISA. NT: Only addition of transfection agent; SSC: side scatter; FSC: forward scatter.

To investigate whether TDP-43 “prion-like” behavior was involved with metabolic disturbances, we performed metabolomics on HEK-293T cells cultured in conditioned medium from other HEK-293T cells having overexpressed TDP-43. Briefly, we overexpressed wild-type (WT) TDP-43 and added the corresponding conditioned medium to naïve recipient HEK-293T cells (Figure 1). Although we did not observe signs of TDP-43 propagation, the naïve cells exhibited tendencies toward lower structural integrity and higher membrane permeability. In addition, these cells demonstrated a metabolome profile that was different from that of untreated cells and cells overexpressing TDP-43, thus suggesting a defect in whole-cell metabolism. These results, altogether, led us to hypothesize that TDP-43-conditioned medium is associated with cellular demise. Furthermore, the molecular environment within TDP-43-conditioned medium is of utmost importance for the understanding of the relationship among TDP-43 propagation, the extracellular environment, and cytotoxicity.

## 2. Materials and Methods

### 2.1. Plasmids

The full-length wild-type human TDP-43 sequence was cloned into the mammalian expression vector pcDNA3.3 (Invitrogen, Strasbourg, France) using the following primers: forward_5′- TCTGAATATATTCGGGTAACCGAAG-3′ and reverse_5′- CTAGTGGTGATGGTGATGATGAGAACCCCCCATTCCCCAGCCAGAAGACTTAG-3′ (Eurogentec, Angers, France). We were interested in the wild-type form of TDP-43 because this is the predominant form found in ALS patients, as mutated TDP-43 accounts for 5% of cases [17]. A histidine tag (6×His) was fused to the C-terminus of wilde-type TDP-43 (wtTDP-43-6×His) to distinguish the overexpressed form from the endogenous form.

### 2.2. Cell Culture and Generation of Conditioned Medium

HEK-293T cells (American Type Culture Collection, Manassas, VA, USA) were the cell line of choice due to its robust transfection efficiency and common application in studies on TDP-43 proteinopathy [11,13,16,18]. We maintained cells in Dulbecco’s Modified Eagle’s Medium (DMEM) supplemented with 5% (*v*/*v*) fetal bovine serum (FBS) at 37 °C (Gibco, Strasbourg, France) and in an incubator maintaining an atmosphere of 5% CO_2_ (Invitrogen, Strasbourg, France). In all, 700,000 cells were seeded in T-25 flasks (Corning, Paris, France). After 24 h, the seeded cells were either transfected with 8 µg of pcDNA3.3-wtTDP43-6×His vector and 16 µL of jetPEI transfection agent (Polyplus-transfection^®^ SA, Strasbourg, France) or treated only with an equal quantity of jetPEI. After 4 h, the growth media were replaced with 8 mL of fresh media, and overexpression was allowed for 72 h. Conditioned medium from TDP-43-transfected cells was referred to as TDP-43 CM and that of cells only treated with jetPEI referred to as NT CM. After 72 h of overexpression, the conditioned media were collected and centrifuged at 1500× *g* for 5 min at room temperature to remove floating dead cells and debris. The media were then added to Amicon Ultra-15 centrifuge tubes (Merck Millipore Ltd., Saint-Quentin-Fallavier, France) and concentrated by centrifugation at 10,000× *g* at room temperature until the volume was reduced 10-fold (V_i_ = 8.0 mL; V_f_ = 0.8 mL), representing a 10-fold concentrated conditioned medium that was then applied to downstream analyses.

### 2.3. Enzyme-Linked Immunosorbent Assay (ELISA) on Conditioned Media

Nunc MaxiSorp 96-well plates (Invitrogen, Strasbourg, France) were coated overnight at 4 °C with 10 µg/mL of anti-TDP-43 antibody targeting the N-terminus (polyclonal rabbit, ProteinTech, Manchester, UK). After 24 h, the coated wells were rinsed three times with phosphate-buffered saline supplemented with 0.1% Tween-20 (PBST) and one time with PBS. Wells were saturated with 4% (*w*/*v*) powdered milk (Régilait©, Saint-Martin-Belle-Roche, France) in PBS (MPBS) for 2 h at room temperature, rinsed with PBST and PBS in the same manner, and incubated with each of the 10-fold concentrated NT CM and TDP-43 CM, and 10 µg/mL of purified GFP-wtTDP-43-6×His (positive control) for 1 h at room temperature. Wells were then rinsed five times with PBST, one time with PBS, and incubated in MPBST with anti-6×His antibody (monoclonal mouse, 1/5000 dilution, ProteinTech, Manchester, UK) for 1 h at room temperature. After rinsing in the same manner, each well was incubated with 100 µL of room-temperature TMB-ELISA solution (Invitrogen, Strasbourg, France) for 5–10 min. Finally, 50 µL of 2 M H_2_SO_4_ were added to stop the reaction, and the absorbance was measured at 450 nm.

### 2.4. Recipient Cell Culture

Recipient cells were either non-transfected or transfected with pcDNA3.3-wtTDP43-6xHis. Each cell type received either NT CM or TDP-43 CM. Non-transfected cells in NT CM (NT CM/NT cells) represented the healthy control. Transfected cells in NT CM (NT CM/TDP-43 cells) represented cells exhibiting TDP-43 proteinopathy in control medium. Non-transfected cells in TDP-43 CM (TDP-43 CM/NT cells) represented cells without TDP-43 proteinopathy in the conditioned medium of cells having endured TDP-43 proteinopathy. Finally, transfected cells in TDP-43 CM (TDP-43 CM/TDP-43 cells) represented cells displaying both TDP-43 proteinopathy and conditioned medium from the same type of cells.

### 2.5. Cell Death, Morphology, and Proliferation

Recipient HEK-293T cells were seeded in 6-well plates (Corning, Paris, France) at 250,000 cells/well in 2 mL of DMEM with 5% FBS. After 24 h, cells were either treated only with 6 µL of jetPEI/well (NT cells) or transfected with 3 µg of pcDNA3.3-wtTDP-6×His vector and 6 µL of jetPEI/well (TDP-43 cells). After 24 h, 200 µL of medium were removed from each well, and 200 µL of 10-fold concentrated NT CM or TDP-43 CM were added to the corresponding wells to dilute the conditioned media to 1×. The rationale was to introduce recipient cells to a 1× concentration of conditioned medium without depriving them of the basic nutrients provided by fresh culture medium. This would control for non-specific effects related to old, nutrient-deprived medium. Following a 24 h incubation at 37 °C, cells were trypsinized in 0.25% trypsin (Gibco, Strasbourg, France), washed in PBS, and resuspended in PBS/5% FBS. For cell death, propidium iodide (Sigma-Aldrich^®^, Lyon, France) was added to samples of 250,000 cells at a final concentration of 10 µg/mL in PBS/1% FBS. This molecule is taken up by dead cells because their plasma membrane becomes less intact. After 30 min of incubation at 37 °C with 5% CO_2_, cells were placed on ice before being analyzed by a Becton Dickinson Accuri™ C6 Plus flow cytometer (BD, Franklin Lakes, NJ, USA). 

Morphology was simultaneously evaluated by sorting cells depending on their side scatter (SSC: granularity) and forward scatter (FSC: size). The distribution of cells from the SSC/FSC ratio of NT CM/NT cells was considered the healthy, morphological control. Densities that deviated from this ratio were considered to have aberrant morphologies.

Proliferation was measured by MTT (3-(4,5-dimethylthiazol-2-yl)-2,5-diphenyltetrazolium bromide) reduction assay (Invitrogen, Strasbourg, France). Recipient cells were seeded in 96-well plates at 10,000 cells/well (Corning, Paris, France) coated with 100 µg/mL poly-D-Lysine (Sigma-Aldrich^®^, Lyon, France) in 200 µL of DMEM with 5% FBS. Replicates of three were seeded per condition. After 24 h, cells were either treated only with 240 nL of jetPEI/well (NT cells) or transfected with 120 ng of pcDNA3.3-wtTDP-6×His vector and 240 nL of jetPEI/well (TDP-43 cells). After a 24 h incubation at 37 °C with 5% CO_2_, 20 µL of medium were removed from each well, and 20 µL of 10-fold concentrated NT CM or TDP-43 CM were added to the corresponding wells to dilute the conditioned media to 1×. Following the 24 h incubation at 37 °C, the medium was removed and replaced with 0.5 mg/mL of MTT diluted in Hank’s Balanced Salt Solution (HBSS, Gibco, Strasbourg, France). Following 30 min of incubation at 37 °C, the medium was withdrawn, the precipitated formazan was solubilized with 100 μL of dimethyl sulfoxide (DMSO; Sigma-Aldrich^®^, Lyon, France), and proliferation was quantified by spectrophotometry at a wavelength of 570 nm.

### 2.6. Immuno-Detection of TDP-43 in Recipient Cells

One million recipient cells in each condition were harvested in ice-cold PBS and centrifuged at 900× *g*. Cell lysis was induced with ice-cold Pierce-RIPA buffer containing protease inhibitor cocktail (Invitrogen, Strasbourg, France) and universal nuclease (Invitrogen, Strasbourg, France) with agitation for 15 min at 4 °C. Protein content of whole-cell lysates was measured by the Lowry method (Bio-Rad, Marnes-la-Coquette, France). Then, 30 µg of protein from each cell lysate were separated in 4–20% SDS-PAGE gel apparatus (Bio-Rad, Marnes-la-Coquette, France) and transferred to PVDF (polyvinylidine fluoride) membranes. After blocking with 5% (*w/v*) Régilait in Tris-buffered saline supplemented with 0.2% Tween-20 (TBS-T), membranes were incubated for 1 h at room temperature with the following primary antibodies: mouse HRP-conjugated anti-β-actin (1/100,000 dilution, ProteinTech, Manchester, UK) for an internal control, mouse anti-GFP (1/5000 dilution, Sigma-Aldrich^®^, Lyon, France) for GFP detection of GFP-6xHis, and mouse HRP-conjugated anti-6×His (1/100,000 dilution, ProteinTech) for detection of GFP-6xHis and wtTDP-43-6×His, respectively. For GFP-specific detection, the primary incubation was followed by 1 h incubation with a secondary antibody coupled to horseradish peroxidase (HRP; anti-mouse, 1/5000; Invitrogen, Strasbourg, France). Chemiluminescence was observed by adding Clarity Western ECL substrate to the membrane followed by visualization using Chemidoc apparatus (Bio-Rad, Marnes-la-Coquette, France) after incubation with ECL. Band intensity was measured with Image Lab software v6.1 (Bio-Rad, Marnes-la-Coquette, France).

### 2.7. Glycolysis and Cellular Respiration of Recipient Cells

Recipient HEK293T cells were seeded in a Seahorse XF96 microplate (Agilent, Paris, France) at 10,000 cells/well in 200 µL of DMEM with 5% FBS. Replicates of three were seeded per condition. After 24 h, cells were either transfected with 120 ng pcDNA3.3-wtTDP43-6×His vector and 240 nL jetPEI (TDP-43 cells) or only supplemented with an equal amount of jetPEI (NT cells). After a 24 h incubation at 37 °C with 5% CO_2_, 20 µL of medium were removed from each well and 20 µL of 10-fold concentrated NT CM or TDP-43 CM were added to the corresponding wells to dilute the conditioned media to 1×. Following a 24 h incubation, the cells were analyzed by a Seahorse XF analyzer (Agilent, Paris, France) to measure the following metabolic parameters: basal respiration, proton leak, maximal respiration, spare respiratory capacity, non-mitochondrial respiration, ATP production, non-glycolytic acidification, glycolysis, glycolytic capacity, and glycolytic reserve.

### 2.8. Metabolomics Analysis of Recipient Cells

A targeted, quantitative approach was employed based on the AbsolutIDQ™ p180 kit (Biocrates, Innsbruck, Austria) using a flow injection analysis and high-performance liquid chromatography (HPLC) tandem mass spectrometry (MS/MS) protocol (Waters, Etten-Leur, The Netherlands). This assay permits the quantification of 188 metabolites [19]. Recipient HEK293T cells were seeded in 6-well plates at 250,000 cells/well in DMEM with 5% FBS. After 24 h, cells were either treated only with 6 µL of jetPEI/well (NT cells) or transfected with 3 µg of pcDNA3.3-wtTDP-6×His vector and 6 µL of jetPEI/well (TDP-43 cells). After 24 h, 200 µL of medium were removed from each well, and 200 µL of 10-fold concentrated NT CM and TDP-43 CM were added to the corresponding wells to dilute the conditioned media to 1×. Following a 24 h incubation at 37 °C, recipient cells were trypsinized and centrifuged at 900× *g* for 5 min at 4 °C. Approximately one million cells were resuspended in 1 mL of ice-cold PBS and centrifuged using the same parameters as above. Cells were dried by removing all of the PBS and then stored at −80 °C. On the day of analysis, cells were thawed and resuspended in ice-cold ethanol/1× PBS at a ratio of 85:15, then subjected to 3 freeze/thaw cycles in liquid nitrogen. Samples were centrifuged at 18,000× *g* at 2 °C for 5 min. Samples were loaded onto a filter paper and dried in a stream of nitrogen for derivation with a solution of 5% phenyl isothiocyanate. Afterward, dried residues were extracted with methanol containing 5 mM ammonium acetate. The MetIDQ^®^ software (Biocrates, Innsbruck, Austria) was used to calculate the concentrations of individual metabolites. Quality controls (QCs) were analyzed regularly on the plate (every 8 samples) to ensure the stability of the mass spectrometer over time.

### 2.9. Statistical Analyses

For metabolomics analysis, two separate analytical methods were employed to identify discriminant metabolites—multivariate and univariate analyses. These analyses were performed using MetaboAnalyst v4.0 (http://www.metaboanalyst.ca). The classification method was based on partial least squares discriminant analysis (PLS-DA). Values of variable importance in projection [20] represent the importance of the metabolite for the PLS-DA models. The score scatter plots show the classified samples, and the loading plot characterizes the relation between the Y and X variables. For each analysis, the standard, default parameters of MetaboAnalyst were applied. The univariate analysis of metabolite levels was based on the fold-change (FC) values and the threshold of significance (*p* < 0.1) after a non-parametric Wilcoxon test (volcano plot), while the multivariate analysis of metabolites depended on the accuracy of the variable importance in the projection (VIP). Therefore, the metabolites that had VIP values > 1 of the multivariate analyses and those that were determined by an FC > 2 (for each comparison) with *p* < 0.1 of the univariate analyses were retained as the most relevant for further discussion [21,22].

Results are shown as mean ± standard error of the mean (SEM). Cytometry results were analyzed using FlowJo VX software v.10.0 (BD Biosciences, San Jose, CA, USA). When relevant, the Mann–Whitney non-parametric *t*-test was performed using Prism v.7.0 (GraphPad Software, San Diego, CA, USA). Results were considered significant with *p* < 0.05.

## 3. Results

### 3.1. Overexpressed TDP-43 Is Released into the Culture Medium 

**Figure 2:**
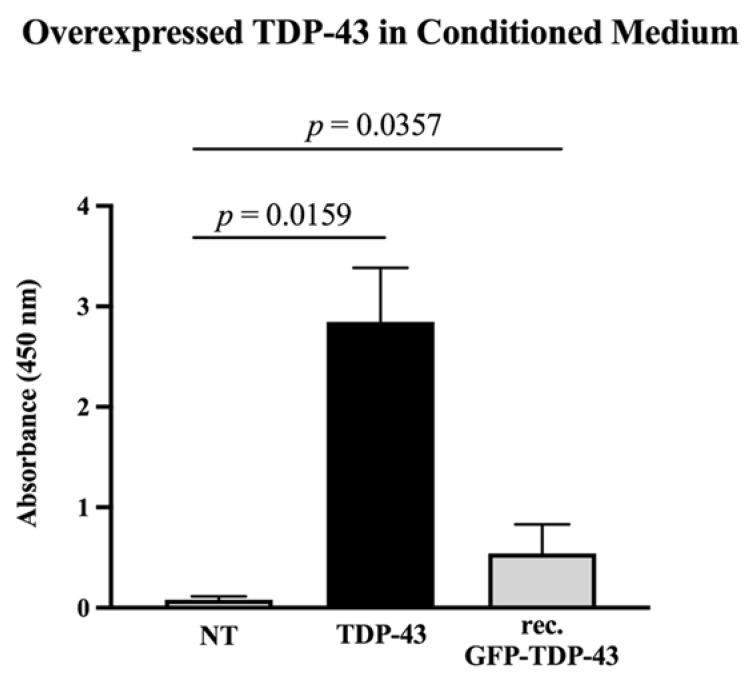
Detecting overexpressed wtTDP43-6×His in conditioned medium. ELISA shows the presence of wtTDP43-6×His in the medium of transfected cells only. The specificity of the antibody used in the sandwich ELISA was validated by incubating with 1.0 µg/mL of purified, recombinant GFP-wtTDP43-6×His that was available in our lab, serving as a positive control. NT: HEK293T only treated with transfection agent; TDP-43: wtTDP43-6×His; rec. GTH: recombinant GFP-wtTDP43-6×His. Statistical test: Mann–Whitney non-parametric *t*-test. *N* = 3–5.

While utilizing an HEK293T cell line overexpressing wtTDP43-6×His (from now on, referred to simply as TDP-43), we observed that overexpressed TDP-43 had been released into the culture medium (*p* = 0.0159) within 72 h post-transfection (Figure 2). The specificity of the sandwich ELISA method employed (Figure 1b) for the detection of overexpressed TDP-43 was validated by the low background absorbance when applying medium from non-transfected cells (Figure 2, white bar) and by the positive absorbance upon applying 1.0 µg/mL of purified GFP-wtTDP43-6×His (*p* = 0.0357) as a positive control (Figure 2, grey bar).

### 3.2. No Propagation of Overexpressed TDP-43

To investigate the involvement of the “prion-like” propagation of TDP-43 in the aforementioned cellular effects of TDP-43-conditioned medium, we performed immunostaining on the lysate of TDP-43 CM/NT cells. However, no sign of internalized TDP-43 was detected following 24 h or 72 h of incubation in conditioned medium. Moreover, longer exposure times of several minutes did not change the result (Figure S1, Supplementary Materials). Therefore, this result suggests that the experimental conditions of this study did not favor TDP-43 propagation.

### 3.3. Increased Proliferation, Increased Propidium Iodide Uptake, and Decreased Membrane Integrity

The proliferation rate of HEK293T cells overexpressing TDP-43 and incubated for 24 h in NT-conditioned medium (NT CM/TDP-43 cells) was significantly reduced (*p* = 0.0286), compared to control (NT CM/NT cells). Conversely, non-transfected cells incubated for 24 h in TDP-43-condtioned medium (TDP-43 CM/NT cells) exhibited a slightly, but significantly, higher proliferation rate (*p* = 0.0286) in comparison to the control (Figure S2A, Supplementary Materials). Furthermore, NT CM/TDP-43 cells showed a significantly altered morphology (*p* = 0.0079) when compared to the control group. A similar alteration was observed in TDP-43 CM/NT cells (Figure S2B), but this did not reach significance (*p* = 0.1270). Regarding cell death by propidium iodide uptake, we did not find significant differences among the conditions but observed the following two notable trends: NT CM/TDP-43 cells tended to reveal a higher amount of uptake (*p* = 0.1), as well as TDP-43 CM/NT cells (*p* = 0.1), compared to NT CM/NT cells (Figure S2C). Altogether, these data suggest that not only TDP-43 overexpression but also TDP-43-conditioned medium could modify the proliferation rate, viability, and structural integrity.

### 3.4. No Change in Oxidative Phosphorylation or Glycolysis

**Figure 3:**
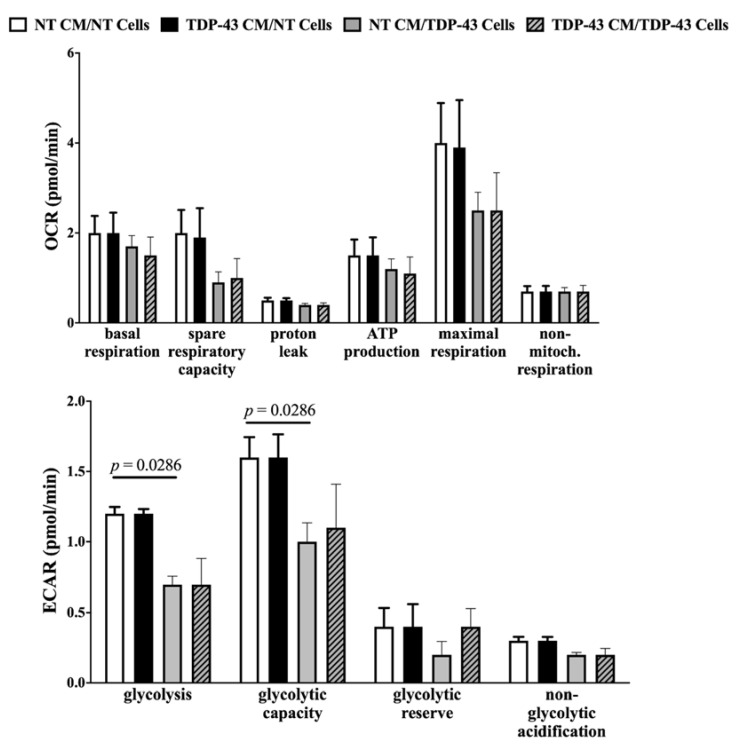
Energy metabolism is modified by TDP-43 overexpression but not conditioned medium. Top: Oxidative phosphorylation represented by oxygen consumption rate (OCR). Bottom: Glycolysis represented by extracellular acidification rate (ECAR). Naïve HEK-293T incubated in TDP-43-conditioned medium did not display significant changes compared to cells incubated in NT-conditioned medium. However, HEK-293T overexpressing TDP-43 in NT-conditioned medium revealed significantly lowered glycolysis and glycolytic capacity. Values represented in pmol/min were normalized to ng of DNA content per mL of sample. Data are presented as mean ± SEM. *N* = 4. Statistical test: Mann–Whitney non-parametric *t*-test.

To assess an effect of TDP-43-conditioned medium on the energy metabolism of cells, the rates of oxidative phosphorylation (oxygen consumption rate (OCR)) and glycolysis (extracellular acidification rate (ECAR)) were measured. Across all metabolic parameters, TDP-43-conditioned medium did not appear to perturb energy metabolism (Figure 3). Neither did TDP-43 overexpression in NT CM/TDP-43 cells seem to affect energy metabolism, apart from diminished glycolytic rate and capacity (*p* = 0.0286). Therefore, these data suggest that neither TDP-43 overexpression nor TDP-43-conditioned medium caused severe alterations in energy metabolism.

### 3.5. TDP-43-Conditioned Medium Caused ALS-Related Perturbations in the Cellular Metabolome

**Figure 4:**
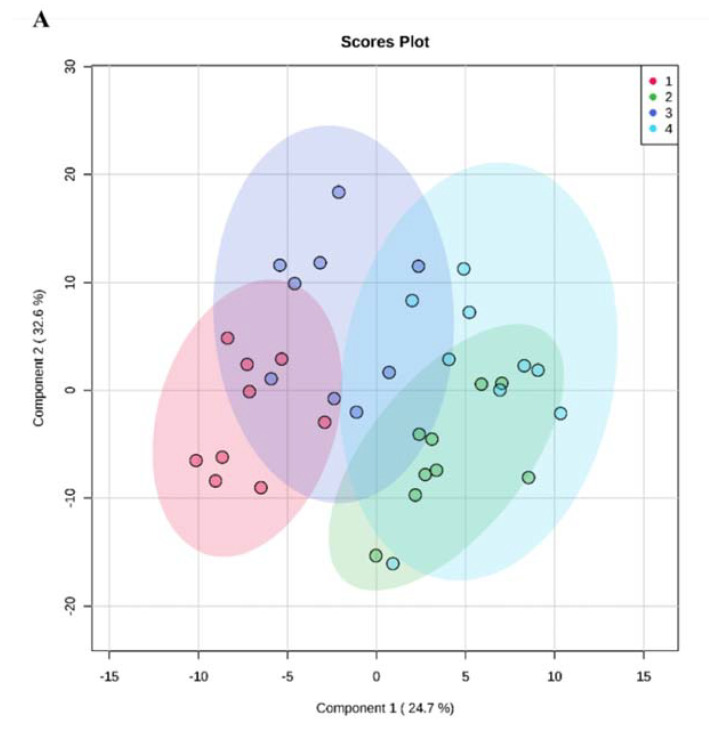

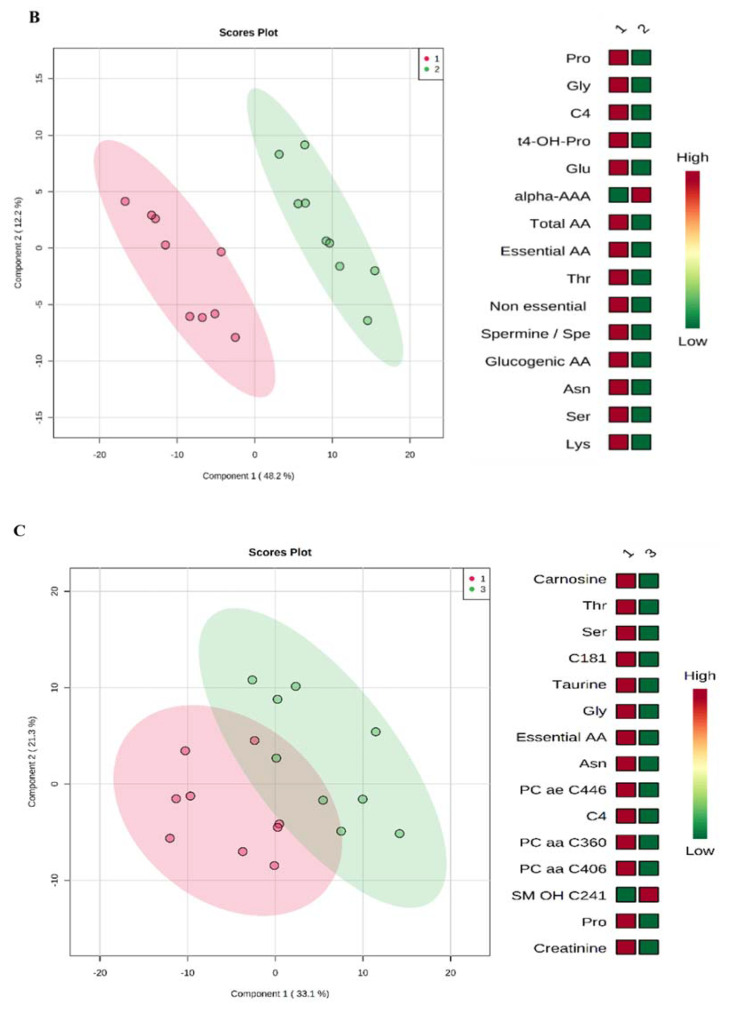

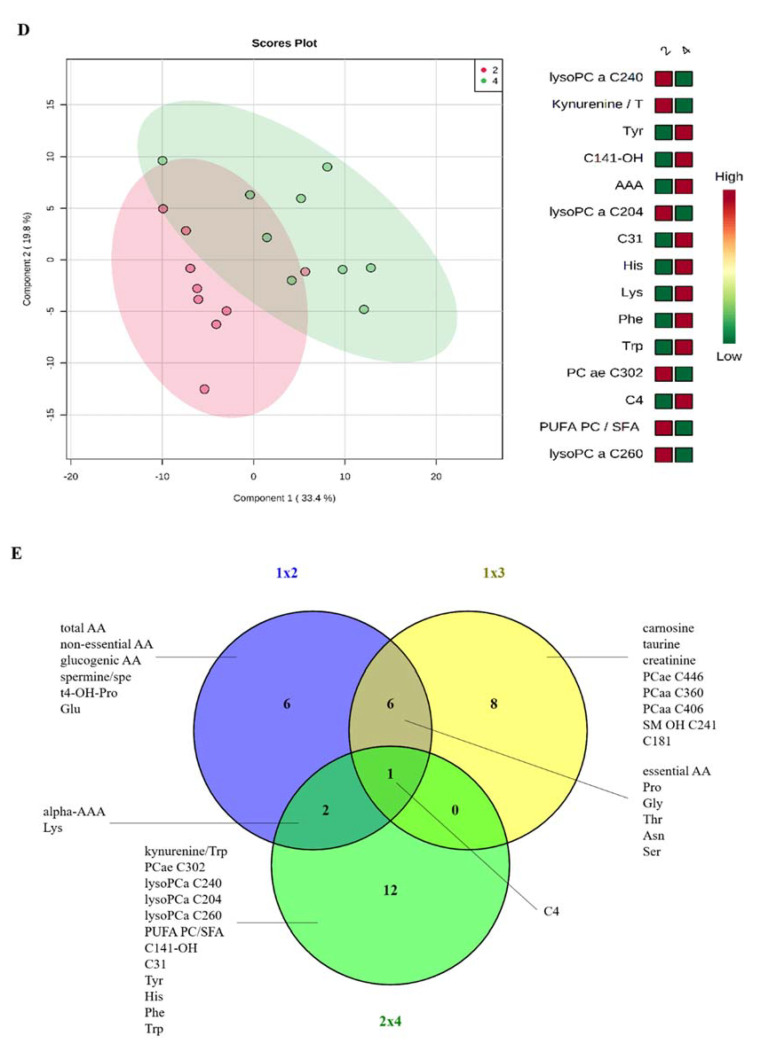
Multivariate analyses of metabolome of HEK-293T in conditioned media. (**A**) Partial least squares discriminant analysis (PLS-DA) scores plot of multivariate analysis of all conditions 1–4 showing differing clusters. 1: NT CM/NT cells, 2: NT CM/TDP-43 cells, 3: TDP-43 CM/NT cells, and 4: TDP-43 CM/NT cells. (**B**) NT CM/NT cells (1) vs. NT CM/TDP-43 cells (2). Left: PLS-DA scores plot. Right: Corresponding top 15 VIP metabolites. (**C**) NT CM/NT cells (1) vs. TDP-43 CM/NT cells (3). Left: PLS-DA scores plot. Right: Corresponding top 15 VIP metabolites. (**D**) NT CM/TDP-43 cells (2) vs. TDP-43 CM/TDP-43 cells (4). Left: PLS-DA scores plot. Right: Corresponding top 15 VIP metabolites. (**E**) Venn diagram of VIP metabolites of all conditions. *N* = 3 for all analyses. Metabolomics analyses were realized with MetaboAnalyst (http://www.metaboanalyst.ca).

Following a multivariate analysis comparing the metabolomes of recipient cells in each condition, we found clusters corresponding to the particular metabolomes of the different conditions (Figure 4A). We can observe that control cells (NT CM/NT cells, red cluster) have a distinct profile of metabolites when compared to TDP-43-overexpressing cells (NT CM/TDP-43 cells, green cluster), to TDP-43-overexpressing cells incubated with conditioned media (TDP-43 CM/TDP-43 cells, blue cluster), and to control cells incubated with conditioned media (TDP-43 CM/NT cells, purple cluster). These observations highlight the metabolic alterations associated with TDP-43 overexpression and/or conditioned media in recipient cells. 

To further identify the most significant metabolites that would explain the separation of the different clusters, we performed 2-by-2 multivariate models for these conditions. First, we investigated the metabolome profile of cells overexpressing TDP-43 in control conditioned medium (NT CM/TDP-43 cells vs. NT CM/NT cells). The top 15 significant metabolites revealed by multivariate analysis (100% accuracy) are displayed in Figure 4B. Second, a multivariate analysis (89% accuracy) was performed on naïve cells cultured in TDP-43-conditioned medium (TDP-43 CM/NT cells vs. NT CM/NT cells). This revealed the 15 most significant metabolites in Figure 4C. Third, the metabolome of cells overexpressing TDP-43 in contact with TDP-43-conditioned medium (TDP-43 CM/TDP-43 cells vs. NT CM/TDP-43 cells) was analyzed by multivariate analysis (78% accuracy). The 15 most important metabolites are shown in Figure 4D.

Finally, the 15 metabolites of each metabolome profile were compared (Figure 4E). Six metabolites appeared to be discriminant only for the model comparing NT CM/NT cells and cells overexpressing TDP-43 (total/non-essential/glucogenic amino acids, spermine/spermidine ratio, t4-OH-Pro, and glutamate). Eight metabolites were discriminant only for the model comparing NT CM/NT cells and naïve cells in TDP-43-conditioned medium (carnosine, taurine, creatinine, PCae C446, PCaa C360, PCaa C406, SM OH C241, and C181). Six metabolites were common between these two models (essential amino acids, proline, glycine, threonine, asparagine, and serine). Twelve metabolites appeared to be only found in the model comparing NT CM/TDP-43 cells and TDP-43-overexpressing cells in TDP-43-conditioned medium (lysoPCa C204/C240/C260, PCae C302, PUFA PC/SFA, C141-OH, C31, kynurenine/Trp ratio, tyrosine, histidine, phenylalanine, and tryptophan). One metabolite was found to be common to all the models presented here (C4).

To determine whether the disturbed metabolites associated with TDP-43 overexpression was a phenomenon associated with the overexpression of whichever protein, we also evaluated the metabolome profiles of TDP-43 and GFP-overexpressing cells without conditioned medium (Figure S3, Supplementary Materials). The metabolome of cells overexpressing GFP was modified compared to the different control conditions and, as shown in the Venn diagrams, the discriminant metabolites were mostly different from that observed in cells overexpressing TDP-43. Therefore, we assumed that the contents of the media from GFP and TDP-43-overexpressing cells were different and influenced by the type of protein overexpressed.

## 4. Discussion

### 4.1. Debatable Findings of Previous Studies on TDP-43 in Conditioned Medium

To our knowledge, two other studies have investigated the effect of TDP-43 in conditioned medium from overexpressing HEK-293T cells, which present contradictory findings pertaining to TDP-43 propagation within conditioned medium. One study indicated the transmission of TDP-43; overexpressed TDP-43 was detected in the medium encapsulated in microvesicles/exosomes and transmitted to recipient HEK-293T and primary neurons, in which cytotoxicity followed [13]. The other study did not show signs of propagation in HEK-293T cells, including its cytoplasmic mislocalization, fragmentation, and phosphorylation. However, lower cell viability was still reported in recipient cells [18]. Due to the apparent cytotoxicity caused by TDP-43-conditioned medium in these previous studies, the goal of the present work was to explore additional cellular effects by TDP-43-conditioned medium, whether or not propagation occurred.

### 4.2. A Fraction of Overexpressed TDP-43 Is Released into the Medium

The recovery of a fraction of the overexpressed TDP-43 in the cell-free medium (Figure 2) corroborated the results from the first study [13]. It is possible that microvesicle/exosome-encapsulated TDP-43 was present in the conditioned media of our study. However, as we only worked with the crude conditioned media, we could not unambiguously determine this. Additionally, since our method of assaying for TDP-43 in conditioned medium did not involve the use of detergents or other chemical treatment to dissolve vesicles, we hypothesized that the TDP-43 detected represented freely released protein from dying cells. This occurrence may represent one mechanism, in addition to exosomes, by which pathological TDP-43 exhibits prion-like behavior [23], which has also been described in the second aforementioned study for misfolded SOD-1 [18]. 

### 4.3. TDP-43 Propagation Did Not Occur by Conditioned Medium

We were unable to observe by immunoblot the propagation of overexpressed TDP-43 in recipient cells incubated in conditioned medium (Figure S1). This could be explained by the fact that the recombinant TDP-43 used by Feiler et al. [13] was fused to two complementary luciferase fragments, which rendered its detection more sensitive than our methods. Their study also included an incubation time of 72 h in conditioned medium, which was longer than that of this study (24 h). Still, the method of this study did not detect internalized TDP-43 after 72 h (Figure S1).

In addition to the factor of assay sensitivity, the state of TDP-43 oligomerization could have influenced the discrepancy between our findings and those of Feiler et al. [13]. Because the structural state of the overexpressed TDP-43 in cells or conditioned media was unknown in our study, it is possible that the form(s) we recovered in the conditioned medium were resistant to propagation. For example, our method of concentrating the TDP-43-conditioned medium 10-fold prior to its addition to recipient cells could have influenced the conformation or oligomerization of TDP-43, which then might have modified its behavior in the medium. In contrast, the overexpressed TDP-43-luciferase construct by Feiler et al. [13] was mostly recovered as a soluble dimer in the cell lysate. Furthermore, the authors apparently did not alter the conditioned medium. Therefore, the soluble dimeric form of TDP-43 from Feiler et al. could have retained propagative behavior, while the form(s) of our concentrated TDP-43-6xHis construct might have lost it.

### 4.4. TDP-43-Conditioned Medium Decreased Structural Integrity, Increased Proliferation Rate and Uptake of Propidium Iodide

HEK-293T cells overexpressing TDP-43 demonstrated compromised morphology, decreased proliferation, and exacerbated cell death, which have been reported in various cellular models of TDP-43 overexpression [4]. Likewise, naïve cells in TDP-43 conditioned medium also experienced a similar trend in altered morphology. The tendency of these cells to take up propidium iodide suggested cell death, reflecting the decreased viability observed in the two similar previous studies [13,18]. However, the unmodified OCR and ECAR of these cells (Figure 3) led us to reconsider the reason for the increased uptake of propidium iodide. Given that it internalizes into dead cells due to their compromised plasma membrane (see Materials and Methods Section 2.5), we hypothesized that the raised propidium iodide uptake could have resulted not from dying cells, per se, but from higher plasma membrane permeability caused by the TDP-43-conditioned medium (Figure S2). The modified glycerophospholipids in TDP-43 CM/NT cells (Figure 4E, 1 × 3) could explain this. Hence, regardless of whether TDP-43 had penetrated naïve recipient cells, cellular defects were induced by TDP-43-conditioned medium.

Remarkably, the conditioned medium seemed to increase proliferation (Figure S2). This could be explained by an activated survival response to manage the stress induced by the conditioned medium. In fact, transcriptomic analyses of post-mortem brain tissue from ALS patients have highlighted both activation of cell death and survival responses [24].

### 4.5. TDP-43-Conditioned Medium and TDP-43 Overexpression Induced Different Metabolome Profiles

Given the different clusters of metabolomes (Figure 4A), we deduced that naïve cells in TDP-43-conditioned medium were associated with a particular metabolome profile in naïve cells. Notably, these cells appeared to show a metabolome with a combination of alterations in common with TDP-43-overexpressing cells and other modifications not found in other conditions (Figure 4E). The common metabolites included essential amino acids, proline, threonine, serine, and asparagine. Indeed, deficiencies in these amino acids were found in a previous study on an ALS cellular model employing SOD-1^G93A^ NSC-34 cells [25]. Interestingly, another group studying a HEK-293T cell model of TDP-43 showed parallel SOD-1 pathology, including its cell-to-cell propagation [18]. Therefore, these highlighted amino acids seem to be associated with both TDP-43 and SOD-1 pathology in the context of ALS. In addition, our study showed that these metabolic disturbances were associated with TDP-43 overexpression and TDP-43-conditioned medium, suggesting a common direct and indirect mechanism of cellular homeostasis alteration.

Furthermore, the eight altered metabolites that were only identified in the model comparing NT CM/NT cells and naïve cells cultured in TDP-43-conditioned medium suggested separate ALS-associated defects not highlighted in TDP-43-overexpressing cells. These metabolites included biogenic amines (carnosine, taurine, and creatinine), glycerophospholipids, hydroxysphingomyelins, and acylcarnitines (Figure 4E). Carnosine and taurine play roles as anti-oxidants within the cell. Indeed, defects in their anti-oxidant properties have been associated with the increased vulnerability to oxidative stress in ALS patients [26,27]. Regarding creatinine, decreased levels have been associated with poor ALS prognosis [28]. The altered glycerophospholipids and hydroxysphingomyelin might reflect defects in the metabolism of these classes of lipids. In effect, the rearrangement of these lipid groups has been observed in ALS patients and SOD-1 murine models [14]. In addition, the modification in hydroxysphingomyelins supported our previous finding that sphingomyelins were generally increased in ALS fibroblasts [29]. Moreover, the highlighted acylcarnitine could reflect the reported defect in the carnitine shuttle in a *Drosophila* TDP-43 overexpression model [15]. As a result, fatty acid beta-oxidation would be perturbed. Indeed, perturbed fatty acid oxidation seems to be linked to the altered lipid metabolism seen in ALS cohorts [30]. These findings support the idea that there is an additional mechanism of cell homeostasis alteration that is not found in cells overexpressing TDP-43 and that merits further exploration by targeted methods.

Interestingly, the effects in biogenic amines, lipid distribution, and acylcarnitines not found in TDP-43-overexpressing cells were then observed in these cells once in contact with TDP-43-conditioned medium (Figure 4E). This suggests a certain synergy of effects between the two conditions. The TDP-43-conditioned medium could further be associated with the cell’s metabolome. For example, the added defect in alpha-AAA metabolism alluded to ALS pathology, as high levels of this biogenic amine in glia have been associated with aberrant glutamate excitatory neurotransmission and astrocytic function [31,32].

Altogether, we observed a metabolism alteration associated with TDP-43 overexpression and TDP-43 conditioned medium with common and distinct discriminant metabolites, suggesting that the putative mechanisms of toxicity are not completely the same. As we showed that these metabolites were not found in the other tested conditions, it will be necessary to show the relationship between these metabolism alterations and cell toxicity and to focus on these alterations, regardless of the specificity to TDP-43.

## 5. Conclusions

The findings of this study suggest that the conditioned medium resulting from cells overexpressing TDP-43 provokes deleterious effects in other cells. These effects seem related to the changes in the metabolome profile of these cells, which could represent the underlying toxic mechanisms. On the one hand, the common metabolome alterations between TDP-43-overexpression and TDP-43-conditioned medium could represent mechanisms that would occur in both cells overexpressing TDP-43 and naïve cells in contact with the associated conditioned medium. On the other hand, the modified metabolites only found in naïve cells with TDP-43-conditioned medium probably indicate distinct and deleterious mechanisms. However, the scope of this study did not allow for the determination of the toxic outcome of such metabolic changes. Therefore, it will be important in future studies to investigate more closely these metabolites to elucidate whether specifically altering them will lead to cytotoxicity. Moreover, future endeavors should include neuronal cell types, such as iPSC-derived motor neurons, to validate these findings.

This study also raises the question as to which species in the conditioned medium acted to bring about such changes. The results shed light on the hypothesis that TDP-43 propagation could not be the only threatening factor in the medium surrounding cells. Since many avenues of the metabolome appeared to be altered in recipient cells, it is unlikely that TDP-43 propagation would be responsible for all outcomes. Furthermore, the release of TDP-43 does not exclude the possibility that other yet-to-be identified species were released in parallel. At this stage, it remains unclear whether the additional factors are specifically linked to overexpressed TDP-43 or non-specifically triggered from the resulting cellular stress. In order to better understand the role of TDP-43 propagation in the context of ALS, these other factors should not be overlooked.

## Supplementary Materials

The following are available online at https://www.mdpi.com/2073-4409/9/10/2198/s1, Figure S1: Immuno-detection of Propagating TDP-43 in HEK293T Cells. Figure S2, Alterations in Cell Functions Induced by TDP-43 Overexpression. Figure S3, Different Metabolomes Result from Different Overexpression Conditions.
