# Supplementary material for: Conditioned Medium from Cells Overexpressing TDP-43 Alters the Metabolome of Recipient Cells
Source: Cells. 2020 Sep 29;9(10):2198. doi: 10.3390/cells9102198 (PMC7601466; doi:10.3390/cells9102198)
Supplement: Supplementary file 1 [file cells-09-02198-s001.pdf]

Supplementary Data

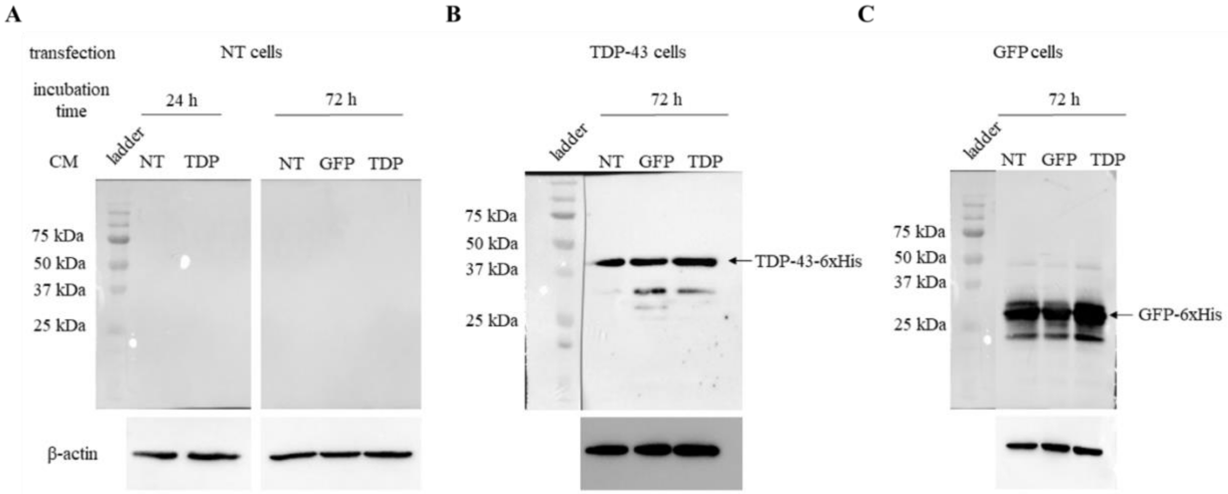

Fig. S1. Immuno-detection of Propagating TDP-43 in HEK293T Cells.

Figure A represents naïve, non-transfected cells incubated in conditioned media for either 24 or 72 h. No His-tagged protein could be detected, meaning that the 72-h incubation period revealed no difference from the 24 h period. Figures B and C show respectively the staining of TDP-43-transfected and GFP-transfected cells, respectively, in each conditioned medium. These conditions served as controls to verify the function of the primary antibodies. Representative images of $N = 3$.

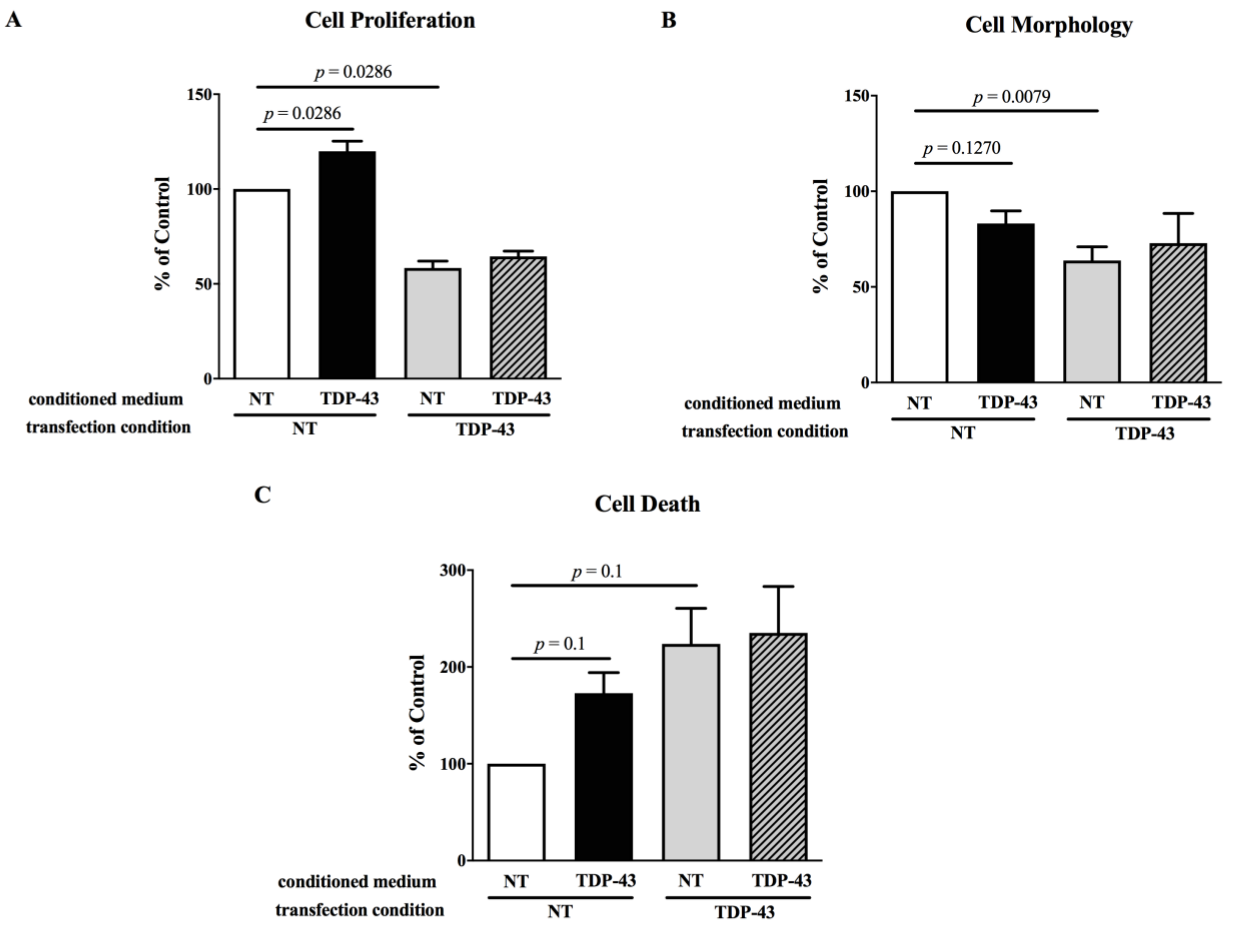

Figure S2. Alterations in Cell Functions Induced by TDP-43 Overexpression.
A) Cell proliferation was measured by the MTT reduction assay. TDP-43 CM/NT cells (black bar) showed a slightly significantly higher rate than NT CM/NT cells (white bar, control). NT CM/TDP-43 cells (striped bar) also demonstrated a significantly lower rate than NT CM/NT cells. N=4. B) Morphological modifications were measured by comparing the SSC/FSC gating among conditions. NT CM/TDP-43 cells (grey bar) showed a significantly lower density of cells with the SSC/FSC distribution of NT CM/NT cells (white bar, control), indicating morphological changes. $N = 5$. C) Cell death was measured with propidium iodide staining. Positive expressing cells represent dead or dying cells that took up propidium iodide. Statistical analysis showed no significance among conditions. $N = 3$. Statistical test: Mann-Whitney non-parametric t-test.

A

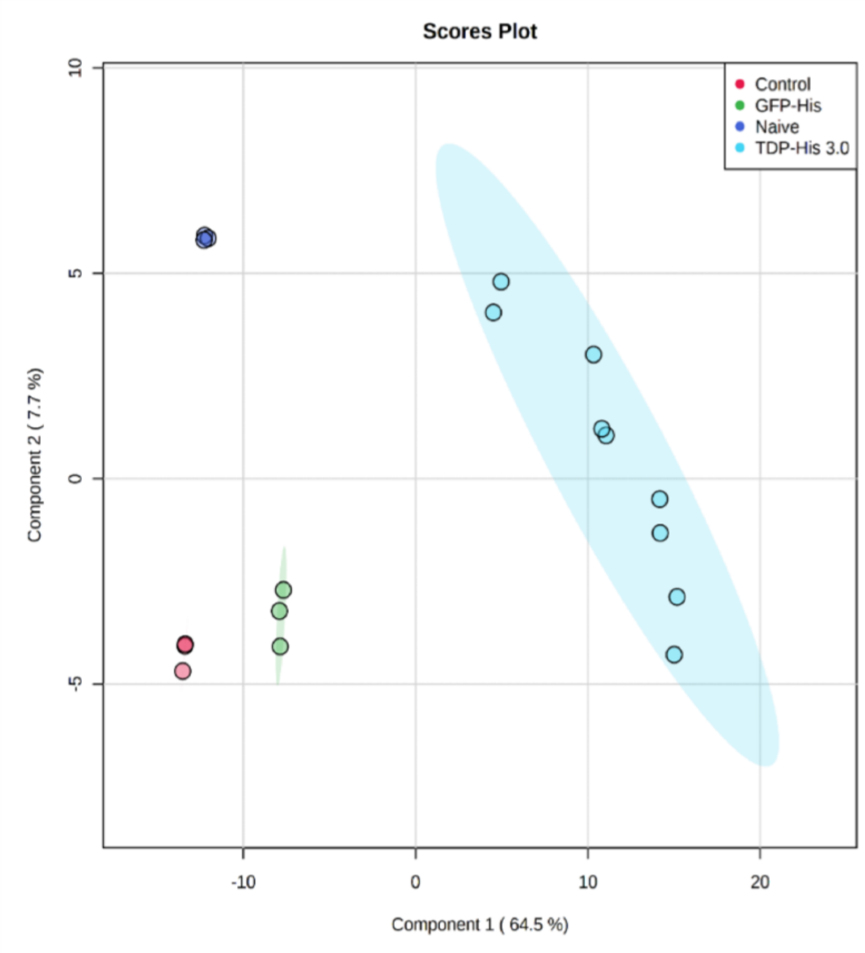

Scores Plot
Control
GFP-His
Naive
TDP-His 3.0
Component 2 (7.7 %)
Component 1 (64.5 %)
10
5
0
-5
-10
0
10
20

B

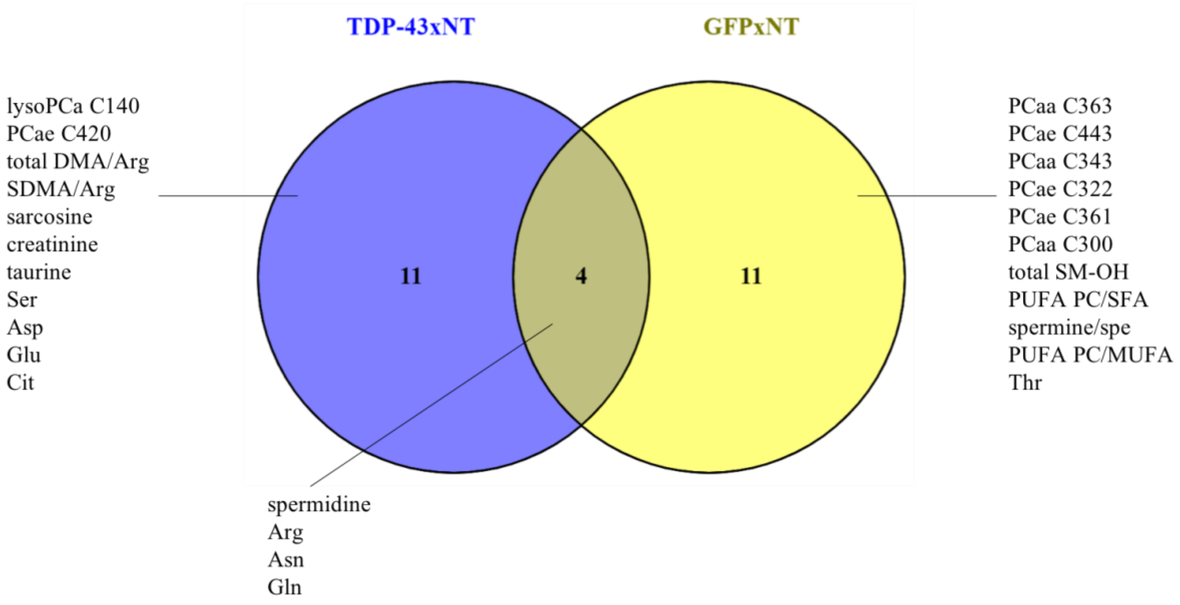

C

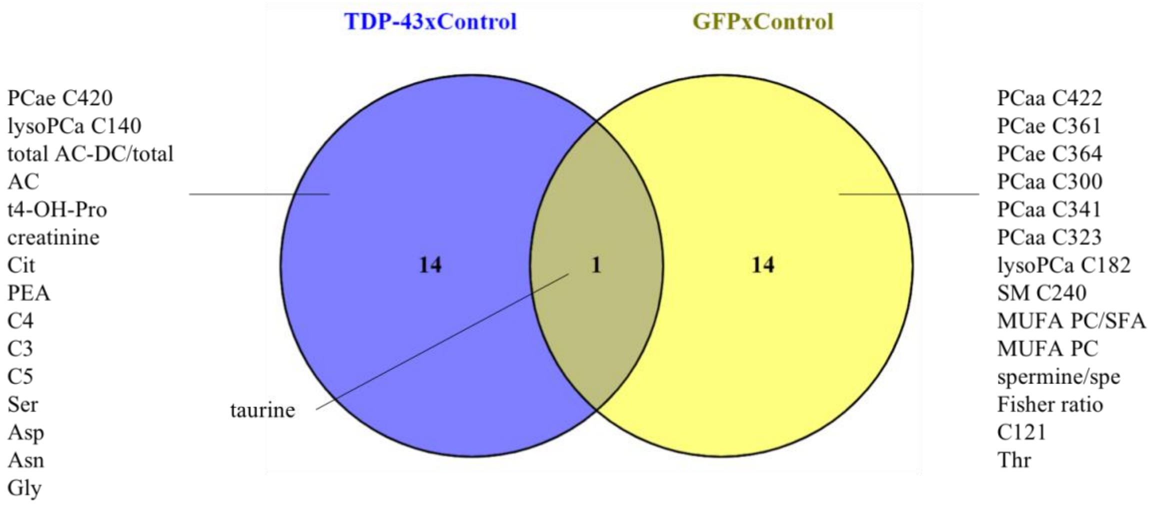

Fig. S3. Different Metabolomes Result from Different Overexpression Conditions
A) PLS-DA scores plot of multivariate analysis. Red: control = HEK-293T cells transfected with 3.0 μg of empty vector. Green: cells transfected with 3.0 μg of GFP-6xHis cDNA. Dark blue: naïve = NT cells only treated with transfection agent. Light blue: TDP-His 3.0 = cells transfected with 3.0 μg of TDP-43-6×His cDNA. B) Venn Diagram comparing the 15 discriminant metabolites from TDP-43 vs Naive and GFP vs Naive conditions. C) Venn Diagram comparing the 15 discriminant metabolites from TDP-43 vs Control and GFP vs Control conditions. No comparison shows the same distribution of metabolites as the other. *N* = 3 for all analyses. Metabolomics analyses were realized with MetaboAnalyst (http://www.metaboanalyst.ca).
